# The Drivers and Impacts of Selling Soil for Brick Making in Bangladesh

**DOI:** 10.1007/s00267-018-1072-z

**Published:** 2018-06-01

**Authors:** Debashish Biswas, Emily S. Gurley, Shannon Rutherford, Stephen P. Luby

**Affiliations:** 1 Infectious Diseases Division, International Centre for Diarrhoeal Disease Research, Bangladesh (icddr,b), Dhaka, Bangladesh; 2 Centre for Environment and Population Health, School of Medicine, Griffith University, Brisbane, QLD Australia; 3 Stanford University, Stanford, CA USA

**Keywords:** Bangladesh, Brick kilns, Crop yields, Food security, Topsoil, Soil degradation

## Abstract

Soil degradation is an important threat to sustainable agriculture. In Bangladesh, brick production contributes to soil loss as the country relies on clay-rich soil for brick making. An in-depth understanding of why farmers sell soil and the corresponding impacts on agricultural productivity is critical for developing and implementing new policies for utilizing alternate materials and methods in Bangladesh and other areas that continue to rely on fired clay bricks as their primary building material. A team of anthropologists conducted 120 structured interviews and 20 in-depth interviews in two different geographical areas in Bangladesh to understand the incentives for and impacts of selling soil. The primary reason farmers sold soil was pressure from neighboring farmers who had previously sold soil. Once neighboring property owners had sold soil, then farmers felt they needed to sell their soil to level their land with the neighboring plot to avoid future production loss. Short-term monetary gain from selling soil was also a strong motivator helping farmers manage acute financial crises. In addition, farmers are frequently compelled to sell soil for brick making because of coercive practices by brick-owners and their soil brokers. In this study, farmers reported 40–80% reduction in crop production and 40–70% reduction in income due to soil removal. The loss of the soil reduces agricultural yields leading to both short- and longer-term impacts on crop production that influence the country’s food security.

## Introduction

Bangladesh’s population growth and increasing affluence have contributed to increased construction and demand for building materials (BBS 2012). Though bricks are the main construction material in Bangladesh, their production, mostly in the form of widely dispersed single small kilns, contribute substantially to poor air quality and poor community health (Brunekreef and Holgate 2002; Cohen et al. 2005; Guttikunda et al. 2013; Ostro 2004; Pope et al. 2002). During the dry season when they operate (normally November to April) they contribute an estimated 40% of the 2.5 micron particulate matter (PM_2.5_) in the air in Dhaka and throughout Bangladesh (Guttikunda et al. 2013; Hossain et al. 2007). Statistical modeling suggests that the air pollution generated by brick kilns results in between 530 and 5000 premature adult deaths annually in Dhaka alone (Croitoru and Sarraf 2012; Guttikunda and Khaliquzzaman 2014).

In addition to air quality problems, brick production impacts agricultural production by utilizing fertile topsoil from agricultural lands (Brunel et al. 2011; Kathuria and Balasubramanian 2013) and releasing toxins, including heavy metals, that influence agricultural productivity (Skinder et al. 2014) and threaten food security (Lal 2013). For example, research in Tamil Nadu, India, found that following topsoil removal for brick production, soil manganese was reduced by 35% and zinc by 63%, and on average, topsoil removal resulted in a loss of about 28 kg of nitrogen, 3 kg of phosphorous and 34 kg of potash per hectare of land. This removal of topsoil was associated with a 124 kg per hectare (3%) reduction in rice yields and 62 kg per hectare (4%) reduction in groundnut yields (Kathuria and Balasubramanian 2013).

In developing countries, soil degradation has been identified as the most important threat to food security (Chen 2007) and in Bangladesh, brick production is one of the main contributors to soil loss (Huq and Shoaib 2013; Khan et al. 2004). The increasing demand for bricks in Bangladesh associated with rapid industrialization and urbanization has raised the demand for soil as a building material. An estimated 5000 brick kilns are currently operating in Bangladesh (World Bank 2011) and most of them are located on fertile agricultural land. Kilns typically occupy 6 acres (2.4 hectares) of agricultural land (Luby et al. 2015) with space required for the firing kiln, storing raw materials and also making and drying green bricks. In aggregate, the brick sector in Bangladesh consumes an estimated 45 million tons of clay every year (World Bank 2011; Guttikunda and Khaliquzzaman 2014). A study conducted in Aligarh in Uttar Pradesh, India, found that over a period of 20 years increased brick production decreased the cultivated area by 8.9% (Singh and Asgher 2005). While the focus of this research is specific to the reasons for soil selling and the consequent impacts on landowners in areas of Bangladesh, it raises the importance of the broader environmental issues relating to the impacts of rapid and often uncontrolled urbanization on prime agricultural land in the vicinity of urban areas. This broader issue is not unique to Bangladesh, or to low- and middle-income countries, with examples provided by the literature from many diverse locations around the world (Bren d’Amour et al. 2016; Satterthwaite et al. 2010).

Despite the use of alternative construction materials and brick designs that reduce the volume of soil inputs used in other countries like China (Chianga et al. 2009; Raut et al. 2011), no such alternative material or designs are currently used in Bangladesh. Other countries including China and several states in India have developed policies towards brick manufacturing including specifying the use of alternative materials. For example, the Indian state Chhattisgarh (population 25 million) has banned clay bricks for any construction and the government has introduced an Environmental Impact Assessment (EIA) requirement for mining of brick making material (Chakravartty 2012; Ministry of Envirionment and Forest MoEF 1998).

The extent of soil removal in Bangladesh is unknown, and only a small number of studies have investigated the effect of soil removal on land productivity (Heierli and Maithel 2008). Better understanding of the system of soil mining to supply brick kilns may identify opportunities for reducing the impact on agricultural productivity. In addition, a more comprehensive understanding of the broader impacts of reliance on clay bricks can inform considerations in Bangladesh and other countries that continue to rely on fired clay bricks as their primary building material. To address this gap in knowledge, this study explored two key areas: (i) why farmers in Bangladesh sell soil from their fertile agricultural land and (ii) the impacts of this soil removal on agricultural productivity and farmer income.

## Materials and Methods

### Study Site and Population

**Fig. 1:**
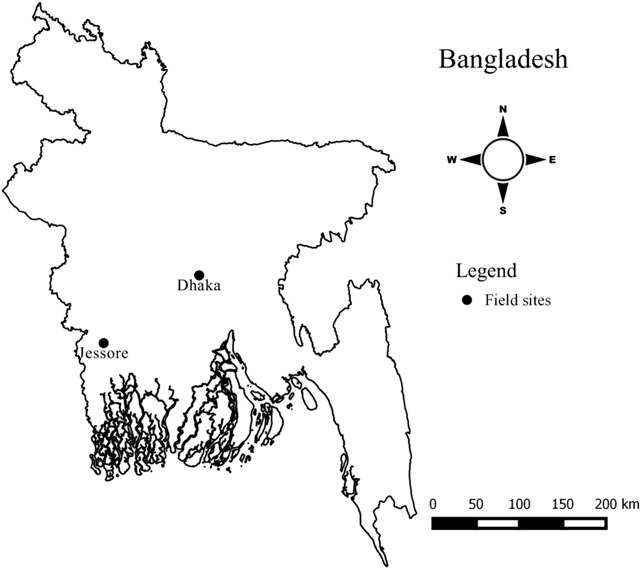
Field sites located near two urban areas of Bangladesh: Dhaka and Jessore

This study was conducted in two districts, Dhaka and Jessore (Fig. 1). These two districts were selected to represent a flood-prone and flood-free area so as to explore the different incentives for soil selling and impact over time due to soil removal. These two types of area were chosen to represent an area that has seasonal flooding (this area generally remains under water for 5–6 months during the rainy season) and one that does not. This is based on the assumption that areas with seasonal flooding would have recharge of topsoil and recovery of agricultural productivity compared with places with no flooding. We selected an area near Dhaka as the flood-prone area because the largest number of brick kilns are located in this area. We selected an area near Jessore as the flood-free area because its elevation is comparatively higher than the flood level and approximately 180 brick kilns operated in Jessore during data collection; a higher number than in the nearby areas. Within these districts we selected a low-lying area located on Brahmaputra flood plain in the West of Dhaka city that was a seasonally flood-prone area, and a flood-free area of Jessore district. A field team collected both quantitative (questionnaire survey) and qualitative (in-depth interview) data between November 2012 to March 2013 and January 2014.

### Survey

To meet the requirements of the quantitative component of the research, our team of anthropologists asked 10 brick kiln owners in each site to identify unions (this is the smallest rural administrative and local government unit in Bangladesh) where they had purchased the most soil for their kilns during the last 10 years. These owners were chosen on the basis of recommendation from the Bangladesh Brick Manufacturing Owners Association (BBMOA). Based on the frequency of responses we selected the two highest ranked unions in each of the two study areas. From each union, 5 villages were randomly selected. Within each village the anthropology team first engaged in several informal conversations with farmers (individually and in a group) in order to list all farmers who sold soil in the past 10 years. We categorized all listed soil sellers into three different groups: farmers who sold soil 1–2 years ago, 3–5 years ago and 6–10 years ago. If a farmer sold soil more than once, we considered the last event of soil selling for categorization. Then, four farmers from within each time frame were selected and interviewed, collecting data for a total of 12 farmers per village, with a target of 60 farmers per union. If an insufficient number of farmers were identified in the randomly selected village, additional farmers from the nearest village were sought. A minimum of 20 farmers were interviewed across villages for each of the soil selling time categories.

We designed a survey to gather data to estimate crop productivity immediately after soil selling as well as estimates of productivity for a number of years afterwards. We used a standard questionnaire developed and pre-tested by the research team at the International Centre for Diarrhoeal Disease Research, Bangladesh (icddr, b) that included questions about how participants had learned of the prospect of selling soil, when and how much soil they sold, how much they earned from selling the soil and estimates of agricultural yield from the year before they sold the soil, the year immediately after they sold the soil and the last year (the year when data collection had taken place) after they sold soil and their income from that particular plot of land where they had sold soil in these three time periods.

Summary statistics were generated from survey data collected from 120 farmers. Pre-and post-selling of soil per plot of land calculations was made for categories of: crop production, area and depth of soil sold, lost agricultural productivity and income. Comparisons of the three time periods associated with the soil sale and agricultural productivity loss and income gain were made. The differences in these variables between the flood-prone and flood-free areas were identified.

### Qualitative Data Collection

For the qualitative component we purposively selected 15 farmers in Dhaka and 5 farmers in Jessore who sold soil from their agricultural land 12–36 months before the date of interview and who were not interviewed for quantitative information.

Anthropologists conducted in-depth interviews with soil sellers to explore the incentives that led them to sell soil, asking how the farmers learned of the prospect of selling soil, how much soil they sold, how much they earned from selling soil, what they did with the money they earned by selling soil and exploring the reasons why they sold soil from their fertile agricultural land. All in-depth interviews were audio recorded. The team also took detailed handwritten notes where needed.

All in-depth interviews were transcribed verbatim, and then summarized and translated into English. They were reviewed and manually coded, relationships between different codes were identified and examined, and then grouped accordingly to summarize the findings from interviews.

## Results

### Soil Removal

The amount of soil sold per farmer was higher in the flood-prone area compared to the flood-free area (Table 1). On average, 877 m^3^ of soil per farmer were removed in the flood-free area compared with more than 3000 m^3^ in the flood-prone area. The mean depth of soil sold was also higher in the flood-prone area.

**Table 1:** Estimated amount of soil sold by farmers for brick making

	Flood-free area (*N* = 60)	Flood-prone area (*N* = 60)
	Mean (95% CI)	Mean (95% CI)
Area (m^2^)	1675 (1225–2125)	2345 (1832–2859)
Depth (m)	0.5 (0.43–0.58)	1.3 (1.04–1.49)
Total soil removed (m^3^)	877 (566–1188)	3380 (1965–4795)

### Drivers for Selling Soil

**Fig. 2:**
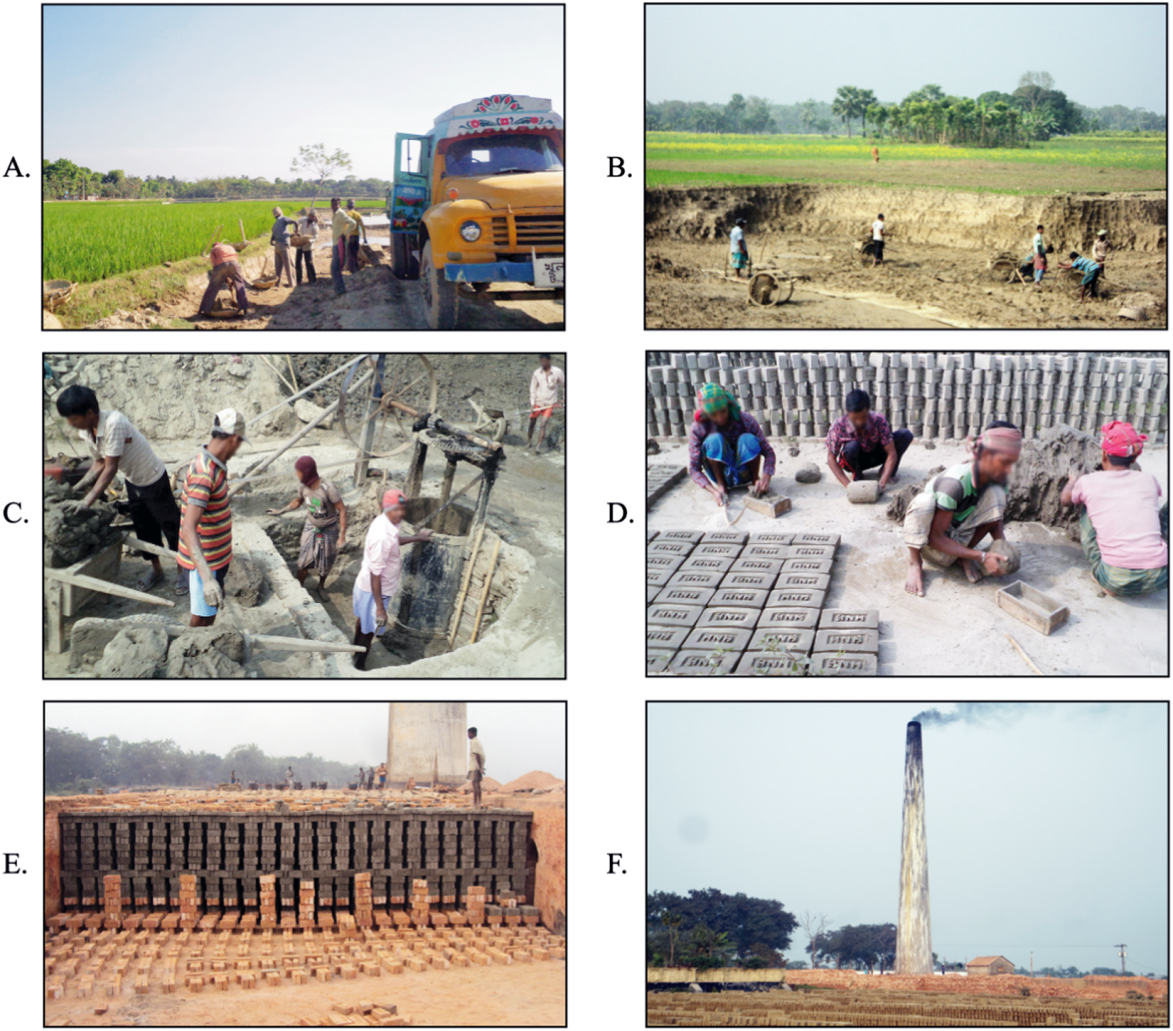
Photo A represents the soil removal from agricultural land; photo B demonstrates the difference between the surface height of the two types of plot—where soil was removed and where soil was not removed; Photos C and D illustrate the brick making process with clay that was collected from farmland; and photos E and F illustrate the brick burning and release of black smoke into the air

**Fig. 3:**
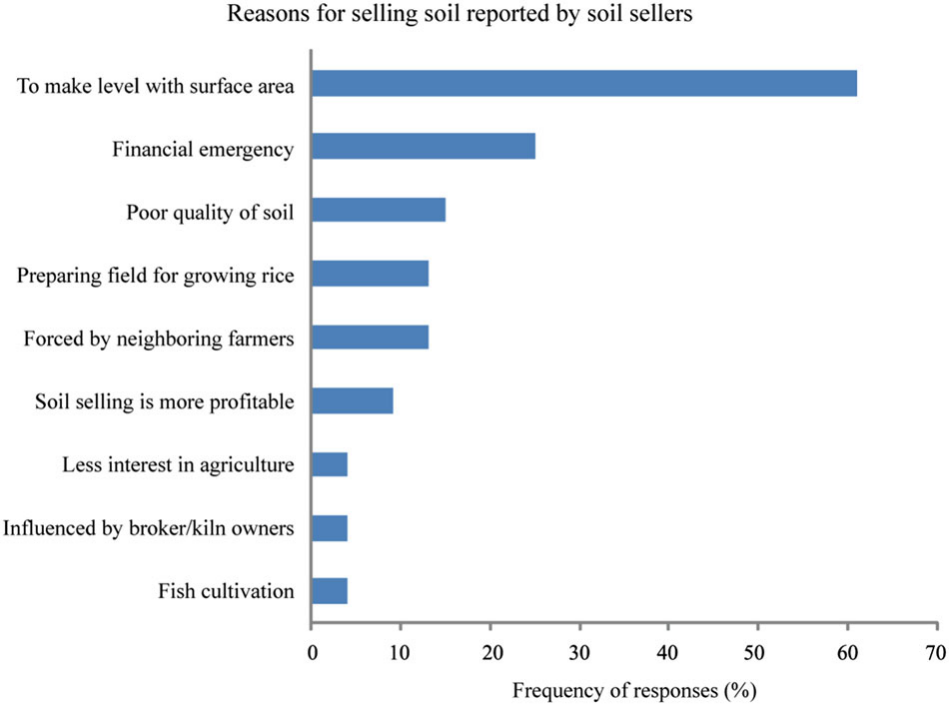
Reasons for soil selling reported by soil sellers (*N* = 120) (multiple responses allowed)

Farmers explained how the increased demand for bricks in nearby fast growing urban areas affected landowners and farmers. The primary reason farmers sold fertile soil from their agricultural land was pressure from neighboring farmers. The removal of soil from neighboring lands changes the local topography and if the farmer does not remove soil from his own land, it is too high relative to his neighbors and it cannot retain water or fertilizer (Fig. 2). This results in markedly reduced productivity and erosion. One farmer explained, *“They (other soil sellers) cut down all three sides beside my land. My land became higher than the nearby lands and thus became unsuitable for cultivation. As a result, I sold soil too”*. This action by neighbors also impacts access to land. For example, one soil seller said, *“My land remained high/dry, but there was water all around the land due to soil removal from those lands, I could not go there easily, also the cattle were not taken there. Therefore, I was forced to sell”*. Thus, 61% (73/120) of the soil sellers reported that they sold soil to make their land level with neighboring plots of land (Fig. 3).

Farmers also explained that sometimes, brick kiln owners themselves purchased land, collecting soil to the maximum depth from that land, hence creating a physical trap that undermined agricultural productivity of adjoining land effectively forcing neighboring farmers to sell their own soil.

The second most reported reason for selling soil was due to a financial emergency (30/120 (25%); Fig. 3). Study participants explained that soil selling is more profitable and has a faster return than growing crops. Further, they do not need to invest any labor or money to earn a return through selling soil. One respondent explained: “*Now the demand and price of soil is high, and rising day by day. I can get more money by selling soil than raising rice”*. Furthermore, when farmers faced a financial emergency and could not secure money from other sources they sold soil from their agricultural land to quickly solve their financial problems. One soil seller said, *“My brother went abroad for a job. In that time we needed a large amount of money urgently, but we could not manage it from other sources. So we sold soil from our land”*. Farmers stressed two issues: firstly, they can earn a large amount of cash at one time without making any investment; secondly, they sell only some soil, not the land, so their land ownership is not affected. For example, one soil seller said, *“I sold just soil from my land, I am not losing ownership. I can do everything in that land and even cropping a few years later”*. Study participants explained that for many farmers or landlords, soil selling has turned into a profitable business. For example, one soil seller explained that, a landowner can earn approximately 100,000 taka (US$1470) by selling 800 m^3^ of soil (the approximate land area is 134 m^2^ or 0.01 hectares). On the other hand, they can produce 650–680 kg of rice on that 0.01 hectares of land in a season if the land is located in a flood-prone area, with a market price of approximately 12,000 taka (US$180). In comparison, they have to wait at least 8 years to earn the equivalent 100,000 taka (US$1470) from soil selling by cropping their land. These interview results are confirmed by survey results presented in the next section. Additionally, farmers’ decisions about soil selling are influenced by uncertainty as to whether they will be able to earn that amount of money through rice cultivation due to climatic events, market price, supply of fertilizers and unfair crop prices.

Farmers explained that soil selling and brick manufacturing leads to new roads and driving across agricultural lands because for brick businesses, transportation facilities are a pre-requisite. They indicated that when trucks/motorized vehicles move beside agricultural lands then these areas are filled by dust which reduces their agricultural production and income. They further mentioned that due to the hot black smoke emitted from the brick kilns, the production rate of crops and fruit in the vicinity decreased, harming the economic productivity of nearby farmers. Participants also reported that low productivity of land motivated them to sell soil. Though their land is cultivable, due to its low production capacity, farmers have less interest in using it for agriculture. For example, farmers explained that in some areas, soil is mixed naturally with sand making that land less productive, particularly for growing rice. In such cases, farmers consider soil selling as the most profitable use of their resource.

Third, soil selling farmers reported that many suppliers or agents were working as middlemen along with brick kiln owners for securing soil supply for brick making. These suppliers and/or agents, commonly known as *dalal* (broker), frequently visit farmland owners pressuring them to sell their soil. For example, one soil seller said, *“Brick kiln owners send some people to us; these are dalal of soil. They tell us, ‘give me some soil, you will get lots of money. Some soil would be sold from your land and you will get huge benefit’*.” In the Jessore field site, farmers indicated that not only the broker, but also the brick kiln owners, encouraged and sometimes forced their employees who own land to sell soil. Study participants reported that brick kiln owners employ farmers on the chance that they can collect soil from them. Participants in the Dhaka area also explained that brick kiln owners sometimes collected soil from their farmland by force. Further to this, sometimes land is stolen and sometimes soil collectors dig soil more deeply without the farmer’s agreement. This happens more frequently to farmers who have limited financial resources and who have no political connections in the community. These examples illustrate the types of coercive behaviors and actions by powerful landlords and brick makers and their agents that exploit poor farmers.

Farmers also described the flooding pattern, acknowledging that the restorative potential of the floods to renew the topsoil was an important factor in their decision making to sell soil. For example, during the rainy season, land in a flood-prone area is filled with flood-water and large quantities of sandy silt are deposited to this land, and thus after a certain period of time following soil removal, the land rejuvenates and fertile soil is replenished.

### Perceived Impact of Soil Removal on Soil Quality and Agricultural Yields

The majority of soil sellers in the flood-prone area (77%) and half of the soil sellers in the flood-free area (50%) perceived that soil quality decreased significantly following soil removal (Table 2).

**Table 2:** Perceived impact of soil removal on soil quality and agricultural yields among farmers who sold soil

Changes in soil quality/agricultural yields	Flood-prone area (*N* = 60)		Flood-free area (*N* = 60)	
	Soil quality freq. (%)	Agricultural yields freq. (%)	Soil quality freq. (%)	Agricultural yields freq. (%)
Soil quality/yields remained the same as previous	5 (8)	2 (3)	5 (8)	3 (5)
Soil quality/yields increased to some extent	–	3 (5)	3 (5)	6 (10)
Soil quality/yields increased significantly	–	–	1 (2)	2 (3)
Soil quality/yields decreased to some extent	9 (15)	8 (13)	21 (35)	23 (38)
Soil quality/yields decreased significantly	46 (77)	47 (79)	30 (50)	26 (44)

Although they identified that initially soil quality decreased following soil removal, during the in-depth interviews, sellers reported that a few years later the land appeared the same as before, due to being filled by silt through flood-water, particularly in flood-prone areas. Farmers also mentioned that soil removal impacts are dependent on the crops grown. Lowland environments were identified as being good for rice cultivation, while vegetables required higher land as soil dries out more quickly. Therefore, removal of soil had little impact on rice yields but significantly reduced productivity of vegetables. Moreover, the impact on crop yields depends on the amount of soil removed. Sand is more prevalent at greater depth and this usually reduces agricultural yields. Therefore, deeper soil removal (more than 0.3 m) sometimes resulted in a drastic loss of soil fertility and the land becoming agriculturally unproductive.

### Impact on Agricultural Production and Income

All soil sellers who cultivated land in the year immediately after they sold soil reported a reduction in crop production compared to the year before they sold soil (Table 3). The differences were larger among the soil sellers in the flood-prone area. In the flood-prone area, farmers produced rice and vegetables such as brinjal, cauliflower, cabbages, bottle gourd, bitter gourd and tomatoes after they sold soil, while farmers in the flood-free area produced more types of crops such as jute, wheat, mustard and lentil in addition to rice and vegetables. In the flood-free area, although rice yields for the most recent year were higher compared to the year before they sold soil, the other crop yields, e.g. vegetables, decreased significantly. Reduction of crop yields in the year immediately following soil removal was 36% in the flood-free area and 77% in the flood-prone area. These crop yields resulted in an average loss of income from crop production of 40% in the flood-free area and 71% in the flood-prone area (Table 4).

**Table 3:** Average reported crop production per hectare in both sites before and after soil removal

Crop	Flood-free area			Flood-prone area		
	The year before soil removal, kg (SD)	The immediate year after soil removal, kg (SD)	The most recent year, kg (SD)	The year before soil removal, kg (SD)	The immediate year after soil removal, kg (SD)	The most recent year, kg (SD)
Rice	6178 (5189)	5535 (4522)	8649 (5189)	4324 (3706)	2718 (4127)	3459 (4596)
Jute	173 (682)	44 (237)	0	3212 (22,042)	0	0
Wheat	104 (593)	49 (366)	49 (366)	0	0	0
Mustard	148 (465)	49 (227)	0	99 (400)	0	0
Lentil	210 (470)	25 (173)	20 (111)	0	0	0
Vegetables	2348 (11095)	141 (1087)	272 (1673)	6054 (11688)	371 (2965)	148 (815)
Total	9161	5843	8990	13689	3089	3607
Decline/increase after soil removal		−36%	−2%		−77%	−74%

**Table 4:** Estimated average income from crop production per hectare of land in both sites before and after soil removal (US$^a^)

Category of soil seller	Flood-free area			Flood-prone area		
	The year before soil removal	The immediate year after soil removal	The most recent year	The year before soil removal	The immediate year after soil removal	The most recent year
Farmers who sold soil 1–2 years previously	2743	1730	2372	3014	1334	1483
Farmers who sold soil 3–5 years previously	2619	1853	4028	4497	1013	1260
Farmers who sold soil 6–10 years previously	3459	1680	3682	3385	815	1334
Average income	2940	1754	3361	3632	1054	1359
Loss/profit per farmer		−40%	+14%		−71%	−63%

^a^US dollars in 2010

A large percentage (70%) of farmers in the flood-free area who sold soil 3–5 years or 6–10 years previously recovered their pre-soil selling agricultural income. In contrast, no soil sellers in the flood-prone area recovered their prior agricultural income even after 6 to 10 years due to the loss in crop yields. On average, farmers in both areas reported that it took at least 15 months to recover their agricultural productivity to those before soil selling. However, they could not cultivate vegetables as productively after the soil removal which disproportionately affected their income.

An examination of estimated net incomes suggests that lost agricultural income following soil selling was compensated for by income from selling soil. The average soil selling earnings were US$1050 per hectare and US$9500 per hectare in the flood-free and flood-prone areas, respectively. One reason for the higher income in the flood-prone area was that there were significant differences in soil price, and a difference in depth of soil sold. This higher price and increased depth may also relate to the area’s proximity to Dhaka, where there is a larger demand for soil and associated bricks due to the rapid urbanization of Dhaka city. We observed an increasing trend over time of earning by soil selling. The average earning by soil selling per hectare of land was higher among the farmers who sold soil most recently (between 1 and 2 years previously) compared to the farmers who sold soil 3–10 years previously (Table 5). Farmers used income from soil selling mainly to meet their daily needs, construction of housing and for health treatments.

**Table 5:** Reported earnings from soil selling

Categories of soil sellers	Flood-free area (US$^a^/hectares)	Flood-prone area (US$^a^/hectares)
Farmers who sold soil between 1–2 years previously	1334	16408
Farmers who sold soil between 3–5 years previously	1013	7240
Farmers who sold soil between 6–10 years previously	815	4843

^a^US dollars in 2010

## Discussion

A fundamental resource for agricultural production in Bangladesh is land, yet agricultural lands are progressively decreasing. In rural areas as well as in areas surrounding urban centers, agricultural lands are increasingly becoming occupied by housing projects or by other business projects (Akther and Hossain 2011; Quasem 2011). In addition to the physical loss of land space for agriculture, the sale of soil for brick making is reducing soil fertility across large land areas and hence threatening agricultural productivity.

This study identified two major factors that influence farmers to sell soil from their agricultural land for brick making and identified coercive practices that further drive soil selling. The number one reason for soil selling is for farmers to keep their land level relative to neighboring lands so as not to lose productivity. This is consistent with that reported in Tamil Nadu, India (Kathuria and Balasubramanian 2013), indicating that it is economically more rational for farmers to sell soil instead of facing the production loss associated with soil removal from neighboring plots.

The second major reason relates to generating income. However, while farmers did earn significant money by selling soil, this income was usually used to meet specific living needs such as daily expenses, housing construction or health treatment and was not commonly reinvested into their livelihood. Household financial crises are a common occurrence particularly for those with low income. Low-income households face many risks and fluctuations in their livelihoods and adopt a variety of mechanisms for coping with anticipated fluctuations (McIntyre et al. 2006). When a household faces a financial shock, selling household assets is a useful and common strategy to make short-term adjustments (Kabir et al. 2000; Sauerborn et al. 1996; Wilkes et al. 1997). In Bangladesh selling soil can help farmers manage acute financial crises, but also they perceive that the short-term financial gain can offset the mid-term loss of agricultural productivity. Moreover, production and price uncertainty are both very common in agriculture (Moschini and Hennessy 2001). Uncertainty in profitability from agriculture and increasing demand and high prices offered by brick kiln owners means that selling soil becomes a viable decision. However, the farmers acknowledge that despite the short-term economic benefits the soil selling process itself leads to development of roads and driving over agricultural lands which in turn can also reduce crop production and associated profit.

Our study confirmed that business owners sometimes coerce low-income farmers to sell soil. This dynamic occurs when people who have money and connections with political parties and have a level of informal power outside of the formal power-base of officials and use such power to benefit themselves at the expense of those less powerful (Devine 2008; Lewis and Hossain 2008). Farmers in Bangladesh are reluctant to take action against such coercive practices of the well-connected kiln owners because of a sense of powerlessness and perhaps a threat of violence that accompanies the unequal power relationships (Lewis and Hossain 2008) or in some cases loss of employment as reported by informants in this research. This coercive dynamic is further compounded by corruption by elected officials (Shah 2006) who have strong connections with unelected elites or may be landowners or kiln owners themselves.

Selling soil has a substantial impact on agricultural productivity and agricultural income. This study estimated a higher loss of productivity compared to the study conducted in Tamil Nadu, India. One reason could be the characteristics of the soil that was more fertile in that region (Kathuria and Balasubramanian 2013). Many factors can affect crop yields following soil removal from fertile agricultural land. For example Oyedele and Aina (2006) explained that topsoil removal primarily effects physical properties and organic matter of the soil, because concentration of organic carbon is higher in the first 15 cm of the soil profile (Bauer and Black 1994). Another study showed that topsoil removal reduces nutritional content of the soil, hence reductions in crop yield (Larney et al. 2000). Although in this study farmers perceived that after a certain period productivity would return to normal, according to their own recollection of yields, farmers in the flood-prone area did not regain their agricultural production even 10 years after selling their soil. Perhaps, one cause could be the depth of soil they removed from their farmland. Other studies have reported similar results; for example, Sui et al. (2009) summarized the relationship between deep soil removal and reduction of crop productivity in a study conducted in China to identify the impact of topsoil removal on corn and soybean yield. Although nutrients can be resupplied to the soil by adding commercial fertilizer the productivity remains affected over time (Lal 2006). High dependency on chemical fertilizers and pesticides to increase crop yields ultimately reduce profit as farmers must spend significant money to buy them (Rasul and Thapa 2004). Hence, removal of a greater depth of soil has both short-term and long-term impacts on crop production influencing food security for the nation. Furthermore, we found that soil removal affected crop choice for cultivation reducing the nutritional value of crop yields and thus influencing food diversity which has adverse impacts on human health (Lal 2009). For example, rice remained planted in both the areas, but cultivation of other crops like jute, vegetables and lentil decreased. In Bangladesh, return generated from rice cultivation did not support improving livelihood of the farmers due to the low profitability of rice compared to other profitable crops such as jute and vegetables previously reported (Hossain et al. 2002). In addition, movement of soil particles during transportation of raw materials for brick production also causes serious problems to human health (Pimentel et al. 2007).

This fertile soil loss in Bangladesh is compounded by other factors that threaten food security. Bangladesh is one of the mostly densely populated countries in the world. Its very high population density has led to a virtually saturated land pattern that has decreased the opportunity to expand agricultural land areas (Streatfield and Karar 2008). Due to the rapid population growth in Bangladesh, rural areas are transforming to developed areas with more infrastructure. Every year about 1% of crop land in Bangladesh is being converted to non-agricultural uses (Hasan et al. 2013). This change decreases the contribution of agriculture to the gross domestic product and contributes to landlessness and food shortage for the country (Ahmad 2005; Dewana and Yamaguchib 2009). At the same time, climate change threatens to reduce agricultural productivity by increasing the risk of floods and droughts and increasing sea-level and associated salinity (Sikder and Xiaoying 2014). Collectively, these issues have led to a deficit in production of food grains in Bangladesh that threaten the country’s food security (Kabir et al. 2016; Hossain and Silva 2013).

There are some limitations in this study. The sample size is small and study subjects were not selected using a method to ensure that they were statistically representative of all soil sellers in Bangladesh, but rather to explore the reasons for soil selling and its impacts from the perspectives of the farmers. We planned sampling to permit us to estimate productivity immediately after soil selling as well as estimates of productivity for periods of years afterwards. Secondly, we selected Dhaka as a study site as it has the largest number of brick kilns and has more options for other works instead of growing crops. However, the impact of soil removal for other flood-prone areas could be different in terms of economic incentives for soil removal due to different soil types, crop prices and brick demand, and studies in other areas would be required to determine if the results here are similar in other locations. Another limitation was relying on recall of the farmers to establish past crop production. However, in the absence of more scientific estimates of crop productivity we believe that these farmers who are directly involved in small-scale farming can provide important information about the impact of soil selling on agricultural production and the income trade-off between selling soil versus selling crops.

Soil is a valuable natural resource for agricultural productivity to ensure sufficient food for the growing Bangladeshi population. As bricks are the main construction material for Bangladesh and there is large and increasing demand for building materials, brick production will continue to grow in the near future and thus the demand for material input will increase. Numerous approaches could reduce this loss of quality soil for agriculture that is critical to Bangladesh’s food security. One option is to use alternative building materials and processes to meet the growing demand for housing. For example, several studies have successfully reported using fly ash to produce bricks which is effective for recycling the industrial waste and can reduce the use of clay (Cultrone and Sebastian 2009; Lingling et al. 2005). However in India, it was found that the use of fly ash actually increases the cost of bricks (Kathuria 2006). A recent study conducted in Bangladesh revealed that people preferred quality bricks at the lowest possible price (Luby et al. 2015). There are some other potential approaches using industrial waste that could be utilized and it would be useful to explore these opportunities by conducting additional research.

Another potential solution is to utilize the existing processes and infrastructure but to conserve the topsoil component, permitting the mining of material below the top layer of nutrient-rich soil. Such changes in practice would require feasibility studies and changes in standard industry practices.

Soil is essential to supply nutritious and quality food for the increasing population of Bangladesh. The present assessment indicates that overall, on balance, current practices of soil mining for brick manufacturing impacts productivity and despite some identified reports of restoration in the Jessore site, this takes around 10 years and results in significant dollars lost. Moreover, farmers are often subject to coercive actions by brick makers and their brokers that gives them little choice in their decision to sell. However, given the importance of diverse agricultural crop production to long-term food security and nutrition in Bangladesh, developing and evaluating approaches that preserve fertile soil for agriculture should be a whole-of-government priority.
